# Association of safety climate with safety performance in pre-hospital emergency health services

**DOI:** 10.3389/fpubh.2025.1624747

**Published:** 2025-10-30

**Authors:** Mehmet Oruç, Rojan Gümüş

**Affiliations:** ^1^Institute of Social Sciences, Dicle University, Diyarbakır, Türkiye; ^2^Atatürk Vocational School of Health Services, Dicle University, Diyarbakır, Türkiye

**Keywords:** pre-hospital emergency, health services, safety climate, safety performance, healthcare workers

## Abstract

**Aim:**

Pre-hospital emergency healthcare workers are often the first responders to critical events. In these situations, they often struggle to comply with safety measures, as they have limited time to save lives while simultaneously ensuring their safety, as well as that of their colleagues and patients. This study aimed to discover the relationship between safety climate and safety performance among pre-hospital emergency healthcare workers.

**Methods:**

The study data were collected from 464 volunteer employees through face-to-face interviews using a personal information form, the Safety Climate Scale, and the Safety Performance Scale. The study performed descriptive statistics, ANOVA, Scheffe tests, student’s *t*-test, regression, and path analyses. It also performed exploratory and confirmatory factor analysis, Cronbach’s alpha, and Skewness and Kurtosis.

**Results:**

According to the results of the study, safety climate (SC) was positively related to the safety performance (SP) of the workers (*β* = 0.480; *p* < 0.001). Those with low safety performance averages were exposed to more violence (*F* = 3.310; *p* < 0.05) and had more occupational diseases (*F* = 2.979; *p* < 0.05) and occupational accidents (*F* = 5.002; *p* < 0.001). While there was no difference in safety climate and safety performance scores between groups in terms of gender and marital status, SC and SP were higher in more educated, older, and higher-income groups. The factors most positively related to SP of employees included awareness and competence (*β* = 0.300; *p* < 0.001), safety communication (*β* = 0.207; *p* < 0.001), and safety training (*β* = 0.163; *p* < 0.001). On the other hand, the organizational environment subdimension of SC was negatively associated with safety performance (*β* = −0.150; *p* < 0.001). As the safe environment in the workplace diminished due to time pressures and uncontrolled scenes, pre-hospital emergency healthcare workers took control and exhibited safer behaviors.

**Conclusion:**

The study findings showed that increased safety climate in the workplace plays an important role in employees’ safe behaviors. Based on these findings, working hours of employees should be regulated; staff numbers, safety training, and safety communication should be increased; and physical conditions should be improved in pre-hospital emergency healthcare.

## 1 Introduction

### 1.1 Pre-hospital emergency health services

Pre-hospital emergency healthcare workers are the first responders to incidents, and their work settings are more dynamic than other healthcare settings. In such settings, they often face challenges in adhering to safety protocols, as they must act quickly to save lives while also ensuring the safety of themselves, their colleagues, and their patients. Pre-hospital emergency medical services aim to provide people with life-saving care at critical moments. As one of the most significant circles of the health sector that provides 24/7 services, it is crucial for public safety and the functioning of the healthcare system (1). The main purpose of the pre-hospital emergency health services, which are also referred to as ambulance services, is to ensure timely medical intervention and support to patients or those injured at the scene of an accident and to transfer them to the hospital rapidly (2).

### 1.2 Pre-hospital emergency health services system in Türkiye

Although emergency health services were first launched in 1920 in Türkiye, pre-hospital health services came into professional action in 1986 in Istanbul, Izmir, and Ankara with the name of Hızır Emergency Service, which operated under the phone number 077. After 8 years, in 1994, the name was changed to 112 Emergency Aid and Rescue. Following this change, the service rapidly expanded to six more cities. A turning point came in 1997 and expanded to the entire country as of that date. In 2003, it operated under the name “Emergency Health Services” (2, 3). The pre-hospital emergency health system in Türkiye predominantly reflects the English-American model, yet the French-German model exists too (4).

### 1.3 Emergency health units in Türkiye

Emergency Health Stations (EHS), the cornerstones of pre-hospital health services, consist of ambulances used in patient transportation and Command and Control Centers (CCC).

#### 1.3.1 Ambulance services

In Türkiye, pre-hospital emergency health services are provided by land, air, and sea ambulances. Land ambulances include emergency aid ambulances, patient transport ambulances, and specially equipped ambulances (pallet ambulances, obese ambulances, and multi-bed ambulances). Air ambulances are aircraft or helicopter ambulances, whereas sea ambulances are vehicles designed for patient transportation over water (3).

#### 1.3.2 Emergency Health Stations

Emergency Health Stations (EHS) are key units within emergency health services. In case of a medical emergency, the 112 ambulance teams that are closest and most suitable to the scene are dispatched from these stations. This system provides 24/7 service free of charge, regardless of geographical situation. Ambulance stations were categorized into three different types (2, 3).

Type A stations are affiliated with the Ambulance Service Chief Physician, have permanent personnel, and provide only ambulance services for 24 h. These stations are classified as A1 if there is a physician in the team and A2 if there is not. Type B stations provide 24 h ambulance and emergency health services within an official health institution/organization. Stations operating within the hospital emergency services are defined as B1, while those offering first-line healthcare are defined as B2. The personnel of type B stations are assigned based on the institution they are affiliated with. The ambulance services provided by the station are managed by the Command and Control Center. Type C stations, however, provide ambulance services at the specified times of the day in line with the requirements (2).

Professionals such as physicians, ambulance emergency care technicians (AECT), emergency medical technicians (EMT), and drivers working in coordination, cooperation, and dedication in prehospital emergency healthcare. These professionals initiate emergency medical care for patients and the injured outside the hospital, maintain this care during ambulance transportation, and complete their duties by delivering relevant information and documents to healthcare professionals in the emergency room. Paramedics, who can also drive the ambulance, prepare for the next emergency case. They hold the title of health technician as an associate degree from two-year colleges or universities (2).

### 1.4 Occupational health and safety in pre-hospital emergency health services

Occupational Health and Safety (OHS) refers to the protection of workers from the negative effects in the workplace and the delivery of a healthy working environment. It also aims to ensure the best harmony between employees and the work and maximize productivity and operational efficiency. Furthermore, it completely prevents or minimizes the risks that may stem from material and moral damage. In short, OHS is the name given to all the attempts to eliminate both short- and long-term health problems likely to occur in the workplace (5). OHS is a public health issue and a crucial problem in terms of worker health in developing countries. Particularly in Türkiye, occupational accidents and occupational diseases are common across all sectors and continue to increase despite other developments in the country (6).

One of the fields with significant risks in terms of occupational health and safety is healthcare services. Healthcare workers are exposed to biological, chemical, physical, ergonomic, and psycho-social risks across various areas of healthcare. The gap existing in occupational health and safety in every aspect of life affects people working in emergency health services more. Besides all these risks, violence against healthcare staff is very common (7).

Ambulance healthcare workers, classified under the high-risk group, face various dangers and risks depending on the nature of their work, as well as the risks faced by employees in other sectors. Due to the nature of their work, they provide healthcare services in unstable locations and under unpredictable conditions, which makes it difficult to take occupational health and safety measures. Ambulance workers and other pre-hospital emergency workers, who spend almost all their working hours in the field, are becoming the most disadvantaged group in terms of occupational health and safety (8, 9).

### 1.5 Risks faced by employees in pre-hospital emergency health services

The risk factors that pre-hospital emergency healthcare workers are most exposed to are physical illnesses, mental problems, sleep disorders, communication disorders, psychosocial illnesses, traffic accidents, occupational diseases, verbal and physical violence, and exposure to chemical and biological agents. These risks and corresponding measures can be categorized in various ways. Among the most exposed ones are ergonomic risks, including musculoskeletal disorders, largely due to heavy lifting. Exposure to ergonomic risks can result in permanent diseases (10).

A significant risk category faced by employees in emergency health services is physical hazards such as tissue injuries, hot-cold weather, and noise (11). Another risk category is exposure to hazardous drugs and substances, as well as chemical risks such as allergic reactions. Biological risks, such as contracting infectious diseases like HIV, hepatitis, and tuberculosis, and contaminated needle sticks, are the most crucial risk categories that emergency personnel are exposed to. In emergency healthcare services, environmental risks such as ambulance accidents, falls, crashes, violence, explosions, and fires are particularly important. Another important risk category for pre-hospital health personnel is psycho-social risks such as stress, anger, loss of motivation, and exposure to mobbing. Other challenges faced by these workers are an unhygienic environment and unhealthy nutrition. The most critical dangers are slipping and falling, object injuries, and muscle injuries due to the emergency nature of their duties (8).

### 1.6 Safety climate

OHS encompasses all efforts aimed at eliminating long, short, and medium term health problems that workers may encounter in the workplace due to the nature of their work. It aims to minimize potential risks in the workplace while reducing or eliminating material and moral damages. In addition, OHS protects employees from negative effects in these settings. It further ensures the best harmony between work and employees. When occupational health and safety increase in a work environment, it maximizes productivity too. To increase occupational health and safety levels in their workplaces, employers should aim to strengthen the occupational safety climate.

Safety climate refers to the perception of the importance of safety in a workplace or an organization over a specific period. These perceptions and beliefs, which can be measured as a value, are shaped by the opinions, actions, attitudes, and values of all employees in the workplace over time and can change with the conditions (12, 13).

According to Zohar (14), one of the first to introduce the concept of safety climate into our lives, safety climate is the overall perception of values that employees attribute to occupational health and safety in an organization at a certain time. Zohar further suggests that the safety attitudes, behaviors, and values of other employees within the workplace can affect employees’ perceptions of safety climate. A high perception of safety climate in the organization can encourage employees to exhibit safer behaviors while increasing productivity by reducing costs. Furthermore, safety climate plays an important role in reducing occupational accidents and diseases (12, 14).

Since safety climate is critical for a workplace, measuring it informs employers about the safety of their workplaces. It also allows for comparison with actual safety performance in the workplace while contributing to strengthening safety efforts in the workplace. Measuring safety climate provides the data and information needed for improvements, changes, and practices necessary for workplace safety. Various scales were developed by different researchers to measure the safety climate in workplaces (13, 15–18). These tools primarily emphasize measuring employees’ perceptions and experiences of safety. While there are differences within each scale, almost all address issues related to the ease of implementing safety procedures, workers’ perceptions of management’s commitment to safety, and the use of safety equipment or tools.

### 1.7 Safety performance

Safety performance aims to minimize incidents and accidents while improving the working environment of workers. Additionally, it saves resources, time, and energy by examining safety systems in place, identifying issues related to safety. It further provides solutions while increasing efficiency and productivity in the workplace. Safety performance encompasses various factors such as safety equipment features, compliance reporting requirements, workforce experience and qualifications, technical specifications and standards, and machine maintenance schedules. Furthermore, it represents behaviors and actions that employees demonstrate in the workplace to promote the safety and health of both them and their customers, the environment, and the general public (16, 19).

Safety performance can be separated into two: “safety compliance” and “safety participation” (13). Safety compliance refers to basic activities, including wearing personal protective equipment, standard operating procedures, and the rules to ensure safety in the workplace, whereas safety participation represents behaviors that do not directly impact employee safety but contribute to the workplace safety environment. These actions include employees’ voluntary behaviors, such as joining in workplace safety activities, attending workplace safety meetings, and assisting colleagues with safety issues. Safety participation and safety compliance differ from each other. Safety compliance refers to a mandatory action regarding the safety rules determined for the employees. Safety participation, on the other hand, includes voluntary and safe behaviors by employees. Safety performance, which combines safety compliance and safety participation, is a key measure of safety in the workplace. If safety performance is measured accurately and effectively, it not only leads to increased workplace safety but also contributes to more efficiency and improved production capacity (13).

Pre-hospital emergency healthcare workers are the first responders to incidents, and their work environment is less stable than other healthcare settings. As mentioned, these employees encounter various risks in the workplace. They sometimes have difficulty complying with safety measures due to the urgency of life-saving interventions, while simultaneously trying to ensure their safety, that of their colleagues, and their patients. Due to the nature of the job, the role of employees is more important than in all other work areas. This points to the importance of teamwork and workplace safety climate in pre-hospital emergency health services. Therefore, the present study aims to determine the impact of pre-hospital emergency healthcare workers’ perception of workplace safety climate on their safety performance.

## 2 Literature review and hypotheses

In workplaces that provide services at an enterprise level, employees’ perceptions of the policies and procedures of management and supervisors, and their implementation in daily practice, constitute employees’ perceptions of the safety climate in a workplace. Literature showed that the safety climate in a company plays an important role in ensuring compliance with procedures and that it enhances employees’ commitment and participation in safety. In line with these studies, theories were developed to create a safety performance model that includes safety compliance and safety participation and is influenced by the safety climate in a workplace (20, 21).

Zohar (14), the pioneer of safety climate studies, was the first to propose the safety climate model to the scientific world. Later, other researchers repeatedly demonstrated in their studies that employees’ perceptions of safety-related policies, procedures, and practices create the safety climate in work environments (16, 20).

Following Zohar, some researchers developed tools to determine the relationship between safety climate constructs and other safety-related variables. The most used and validated safety climate constructs include perceptions of safety communication, safety training, safety systems and procedures, and management commitment to safety (17, 18, 20, 22).

Although the sub-dimensions of safety climate are specified differently in many studies, the positive effect of safety climate on safety behaviors and outcomes was well researched, and its impact on factors such as accidents, near misses, or injuries has been demonstrated (23, 24).

A meta-analysis by Clarke (25) combined 35 studies on the effect of safety climate on safety performance. This meta-analysis, which combined many studies, found strong correlations between safety climate and safety compliance and participation. Furthermore, the positive effect of safety climate on employee safety performance was found in many other safety climate studies (16, 23, 26).

Some previous work conducted with healthcare professionals reported a significant relationship between the workplace safety climate and employee safety performance (24, 27, 28). A study conducted with 211 nurses working in three hospitals in Iran identified that safety training and managerial attitude, which are among the sub-dimensions of safety climate, had the greatest impact on safety performance (29). The same study indicated that there was a positive relationship between safety participation and the perception of safety climate.

McGhan et al. conducted a study of 221 healthcare workers in Canada in 2020 (30). The study found that healthcare workers with low perceptions of safety performance exhibited lower safety behaviors and therefore experienced more occupational injuries. In the same study, safety training, one of the sub-dimensions of safety climate, was found to be the most significant factor in safety performance.

Alghalban et al. (31) studied the effect of safety climate on work accidents among 350 health workers in primary and secondary health services in Egypt and reported that safety communication and use of safety equipment were the most important factors on employee safety performance. Cook et al. (32) in the United States and Agnew et al. (33) in Scotland conducted research with a very large sample of healthcare workers and reported that the perception of the safety climate in the hospital was very important for the safe behavior of employees.

Considering the previous literature, it is obvious that the safety climate in a workplace (management support, awareness and competence, risks and precautions, safety communication, organizational environment, safety training) is related to employees’ safety compliance and participation. Based on the previous literature, the following hypotheses were generated:

*H_1_:* There is a significant relationship between perceived safety climate and safety compliance of pre-hospital emergency healthcare professionals.

*H_1a,1b,1c,1d,1e,1f_:* There is a significant relationship between perceived safety climate (a. awareness and competence, b. safety communication, c. organizational environment, d. management support, e. risks and precautions, f. safety training) and safety compliance of pre-hospital emergency healthcare professionals.

*H_2_:* There is a significant relationship between perceived safety climate and safety participation of pre-hospital emergency healthcare professionals.

*H_2a,2b,2c,2d,2e,2f_:* There is a significant relationship between perceived safety climate (a. awareness and competence, b. safety communication, c. organizational environment, d. management support, e. risks and precautions, f. safety training) and safety participation of pre-hospital emergency healthcare professionals.

*H_3_:* There is a significant relationship between perceived safety climate and safety performance of pre-hospital emergency healthcare professionals.

## 3 Methods

### 3.1 Procedures and participants

This research is a cross-sectional study, and the data were obtained through face-to-face interviews conducted with employees working in pre-hospital emergency health services in the center of Diyarbakır, Türkiye. The universe of this research consists of 778 individuals who were actively working in pre-hospital emergency health services in Diyarbakır city center during the period of the research. The appropriate sample size was calculated using the formula (*n* = (Nt^2^pq)/(d^2^ (*N*−1) + t^2^pq)) where the number of potential research groups is known. The required minimum sample size was found to be 384 employees with a confidence level of 95% and a margin of error of 0.05. The data in the research were collected from 70 emergency health services, emergency health administration units, and command control centers in Diyarbakır city center between February 2024 and June 2024. No sampling method was used in the research, and an attempt was made to reach all employees. The vast majority of surveys were conducted face-to-face, but few questionnaires were left at stations only for employees who were on-site for the incident and those working night shifts but were not in the buildings at the time. All the participants were asked to complete the questionnaires without pressure. The surveys were conducted by the researchers and volunteer colleagues. After removing invalid surveys, statistical analyses were performed on the remaining 464 surveys. The number of valid surveys obtained represents 60% of the research universe.

### 3.2 Including and excluding criteria

Inclusion criteria for the study were to work in pre-hospital emergency care, to be actively working during the study period (those on leave and those reporting were excluded), and to have volunteered for the study.

### 3.3 Ethical aspects

Before starting the research, an application was made to the Diyarbakır Provincial Health Directorate and permission for the study was obtained (date: 16.01.2024, number: E-90410089-799-234268788). The participants who completed the questionnaires were informed about the study, and written consent was obtained from all participants. Ethics committee approval was obtained before the research (Dicle University Ethics Committee, date: 09.11.2023, number: E-14679147-663.05-597556) and the study was conducted according to the Declaration of Helsinki. Permission was obtained from the authors who adapted the scales used in the study to the Turkish language before the research.

### 3.4 Measurement tools

A three-part questionnaire was administered to the participants. In the first part of the survey, a personal introduction form consisting of socio-demographic questions was used, and in the second part, a safety climate perception questionnaire consisting of 21 items and 6 dimensions developed by Lin et al. (15) and adapted to Turkish by Deveci et al. (34) was used. The adaptation of the scale to the Turkish language was carried out based on standard international methods. To ensure the language validity of the scale, two English language experts whose native language is Turkish translated the scale items into Turkish. The items were then discussed with the translators and experts from various disciplines. The final version of the scale was translated from Turkish to English. All items were then translated back into Turkish by a third language expert. Following a pilot study with ten participants, psychometric adaptation of the scale began. The authors declared that the Turkish version of the Safety Climate Scale was high in explanatory and internal consistency in terms of psychometric properties and has a good model fit. Deveci et al. found that Cronbach’s alpha value for the entire scale was 0.879. An increase in the score to be obtained from the scale indicated high importance for the relevant item.

In the third part of the survey, a safety performance questionnaire consisting of 8 items and 2 dimensions, developed by Neal et al. (12) and adapted to Turkish by Sakallı et al. (35), was used. Both scales consist of five-point Likert-type questions. The five-stage technique proposed by Brislin et al. (36) was used in the Turkish translation study. These stages included initial translation, evaluation of the initial translation, back translation, evaluation of the back translation, and expert opinion. The English version of the scale was translated into Turkish by three Psychology and Occupational Health and Safety experts fluent in both English and Turkish. The English translation was then based on these Turkish translations and compared with the original scale to complete the back-translation process. Sakallı et al. found that the Cronbach’s alpha coefficient for the adapted “Safety Climate Scale” was 0.970.

### 3.5 Data analysis

In this study, IBM SPSS 30 and IBM AMOS 30 programs were used to perform path analysis, descriptive statistics, skewness and kurtosis values, Cronbach’s alpha reliability coefficient, EFA and CFA analyses. One-way ANOVA and Scheffe test, Student’s *t*-test, were used to compare safety climate perception and safety performance scores for all socio-demographic groups. To determine the relationship between subdimensions of safety climate and safety performance of the employees Path Analysis and Regression Analysis were used. For all analyses, a p-level of <0.05 was considered significant.

## 4 Results

### 4.1 Exploratory factor analysis, confirmatory factor analysis, and homogeneity tests of safety performance and safety climate constructs

To underline the structure of the constructs and subsequently apply further analysis as Regression and Path Analysis, an Exploratory Factor analysis (EFA) using principal components analysis with varimax rotation was used. Cronbach’s alpha values, homogeneity test results, means, and standard errors, EFA results for each construct of pre-hospital healthcare workers can be found in Table 1.

**Table 1:** Summary of Cronbach’s alphas, means, standard errors, EFA results and homogeneity test results for each construct.

Construct/factor extracted	KMO	Bartlett	Cumulative variance (%)	Cronbach’s alpha	Mean	SE	Min–max	Skewness	Kurtosis
Safety climate/6 factors	0.871	χ^2^ = 4038.469 *p* < 0.001	70.863	0.889	3.184	0.563	1–5	−0.144	−0.270
Safety performance/2 factors	0.865	χ^2^ = 1849.272 *p* < 0.001	68.484	0.878	4.111	0.657	1–5	−0.777	1.292

KMO, Kaiser-Meyer-Olkin; EFA, exploratory factor analysis.

Looking at Table 1, the Cronbach alpha values were well above the recommended level of 0.700, indicating sampling adequacy (37). The safety climate and safety performance constructs showed good skewness and kurtosis values, allowing parametric tests to be applied to the dataset.

When confirmatory factor analysis (CFA) was applied, all goodness of fit indices for safety performance and safety climate were found to be within acceptable ranges. Table 2 shows a summary of the confirmatory factor analysis for the constructs.

**Table 2:** Summary of confirmatory factor analysis results for constructs.

Indices	Model Fit Index	Levels of acceptable fit	Safety performance	Safety climate
χ^2^	x	x	336.527	57.911
df	x	x	132	17
χ^2^/df	≤3	≤5	2.255	3.407
GFI	≥0.95	≥0.90	0.927	0.970
AGFI	≥0.90	≥0.85	0.895	0.937
CFI	≥0.97	≥0.90	0.945	0.978
RMR	≤0.05	≤0.08	0.057	0.028
RMSEA	≤0.05	≤0.08	0.058	0.072

RMSEA, Root Mean Square Error of Approximation; χ^2^/df, Chi-Squared/degree of freedom; AGFI, Adjusted Goodness of Fit Index; CFI, Comparative Fit Index; RMR, Root Mean Residual; GFI, Goodness of Fit Index.

As shown in Table 2, the indices CFI, χ^2^/df, GFI, RMSEA, AGFI, and RMR indices confirmed the unidimensional of the model constructs and were found to be satisfactory.

### 4.2 Safety performance and safety climate perception scores, socio-demographic characteristics, and other factors

Table 3 demonstrates the safety performance and safety climate perception scores of healthcare workers based on sociodemographic characteristics and some other factors.

**Table 3:** Safety performance and safety climate perception scores, socio-demographic characteristics and other factors.

Variable		*n*	%	Safety climate	Safety compliance	Safety participation	Safety performance
Sex	Female	278	59.9	3.211 ± 0.550	4.042 ± 0.800	4.254 ± 0.652	4.140 ± 0.672
	Male	186	40.1	3.143 ± 0.572	3.890 ± 0.790	4.233 ± 0.684	4.061 ± 0.642
	*t*			1.373	1.920	0.342	1.336
	*p*			0.171	0.056	0.732	0.182
Age	18–24^a^	41	8.0	3.301 ± 0.460	3.972 ± 0.741	4.184 ± 0.772	4.072 ± 0.690
	25–29^b^	208	44.8	3.143 ± 0.543	3.941 ± 0.810	4.304 ± 0.650	4.123 ± 0.651
	30–34^c^	91	19.6	3.071 ± 0.552	3.923 ± 0.781	4.172 ± 0.623	4.042 ± 0.633
	35–39^d^	75	16.1	3.232 ± 0.632	4.034 ± 0.823	4.193 ± 0.663	4.112 ± 0.681
	+40^e^	49	10.5	3.463 ± 0.503	4.312 ± 0.750	4.243 ± 0.742	4.283 ± 0.734
	*F*			4.207	1.853	0.841	0.829
	*p*			**<0.01**	0.118	0.500	0.507
				**e > b, e > c**			
Marital status	Married	290	62.5	3.180 ± 0.562	4.033 ± 0.787	4.223 ± 0.644	4.122 ± 0.663
	Single/Divorced	174	37.5	3.180 ± 0.543	3.912 ± 0.814	4.272 ± 0.689	4.088 ± 0.663
	*t*			−0.108	1.578	−0.827	0.539
	*p*			0.914	0.115	0.408	0.590
Education level	High School	96	20.7	3.273 ± 0.566	4.132 ± 0.833	4.232 ± 0.765	4.177 ± 0.753
	Vocational School	164	35.3	3.122 ± 0.523	3.888 ± 0.785	4.232 ± 0.623	4.061 ± 0.614
	Undergraduate	159	34.2	3.158 ± 0.578	3.959 ± 0.788	4.223 ± 0.654	4.088 ± 0.64
	Postgraduate	45	9.6	3.32 ± 0.54	4.16 ± 0.81	4.42 ± 0.63	4.29 ± 0.65
	*F*			2.249	2.268	0.797	1.420
	*p*			0.082	0.080	0.496	0.236
Monthly income (TL)	35,000–40000^a^	106	22.8	3.263 ± 0.644	4.112 ± 0.833	4.312 ± 0.622	4.214 ± 0.649
	40,001–45000^b^	174	37.5	3.192 ± 0.532	3.955 ± 0.772	4.191 ± 0.654	4.078 ± 0.643
	+45000^c^	184	39.6	3.166 ± 0.543	3.94 ± 0.77	4.320 ± 0.59	4.13 ± 0.6
	*F*			1.945	1.426	2.677	1.869
	*t*			0.122	0.234	**<0.05**	0.134
						a > b, c > b	
Work experience (years)	<5	141	30.4	3.243 ± 0.522	4.001 ± 0.822	4.301 ± 0.577	4.150 ± 0.621
	6–10	174	37.5	3.110 ± 0.530	3.960 ± 0.765	4.230 ± 0.699	4.087 ± 0.667
	11–16	74	15.9	3.201 ± 0.61	3.910 ± 0.799	4.190 ± 0.621	4.050 ± 0.621
	>16	75	16.2	3.221 ± 0.632	4.051 ± 0.824	4.201 ± 0.740	4.132 ± 0.741
	*F*			1.628	0.478	0.570	0.418
	*t*			0.182	0.698	0.635	0.740
Working hours	24 h	415	89.4	3.159 ± 0.553	4.071 ± 0.889	4.100 ± 0.862	4.079 ± 0.842
	Other	49	10.6	3.332 ± 0.596	3.969 ± 0.788	4.262 ± 0.631	4.112 ± 0.634
	*t*			2.055	0.841	−1.586	−0.286
	*p*			**<0.05**	0.401	0.113	0.775
Number of occupational accidents in the last 2 years	None^a^	245	52.8	3.264 ± 0.562	4.078 ± 0.810	4.251 ± 0.661	4.172 ± 0.668
	1–3 times^b^	180	38.8	3.112 ± 0.533	3.849 ± 0.761	4.23 ± 0.64	4.04 ± 0.62
	More than 3 times^c^	39	8.4	2.7 ± 0.48	3.82 ± 0.78	4.142 ± 0.890	3.978 ± 0.761
	*F*			11.095	5.002	0.288	2.435
	*p*			**<0.001**	**<0.01**	0.750	0.089
				a > b, a > c	a > b, a > c		
Number of occupational diseases in the last 2 years	None^a^	158	34.1	3.369 ± 0.521	4.132 ± 0.741	4.321 ± 0.581	4.221 ± 0.568
	1^b^	132	28.4	3.177 ± 0.490	4.001 ± 0.800	4.241 ± 0.702	4.121 ± 0.678
	2^c^	122	26.3	3.051 ± 0.5623	3.841 ± 0.787	4.151 ± 0.663	3.989 ± 0.662
	More than 3 times^d^	52	11.2	2.879 ± 0.591	3.821 ± 0.896	4.231 ± 0.821	4.021 ± 0.788
	*F*			14.751	3.993	1.183	2.979
	*p*			**<0.001**	**<0.01**	0.316	**<0.05**
				a > b+, a > c+, a > b+	a > d		a > c
Exposure to violence in the last 2 years	None^a^	138	29.7	3.401 ± 0.511	4.102 ± 0.755	4.321 ± 0.611	4.212 ± 0.612
	Verbal and physical	216	46.6	3.143 ± 0.542	4.002 ± 0.787	4.200 ± 0.731	4.111 ± 0.699
	Verbal. physical and psychological	110	23.7	2.971 ± 0.552	3.7821 ± 0.82	4.2132 ± 0.58	4.002 ± 0.623
	*F*			21.459	5.045	1.659	3.310
	*p*			**<0.001**	**<0.01**	0.192	**<0.05**
				a > c+	a > c+		a > c+

*t*, student’s *t* test; F, anova test; +, post-hoc analysis; scheffe test.

Bold values describe the significance of *p*-level based on <0.05.

Based on dependent *t*-tests and ANOVA tests, and Scheffe tests, while employees over 40 reported a higher perception of the safety climate in the workplace (*F* = 4.207; *p* < 0.001), younger groups (25–33, 38) who were assigned to the most hazardous front-line roles rated the safety climate of their workplace lower. In addition, the means of safety climate perceptions were significantly different according to the working hours of the employees. Participants working 24 h shifts (89.4%) reported lower mean safety climate scores (t = 2.055; *p* < 0.05). When income status was considered, respondents from higher (39.6%) and lower income groups (22.8%) had higher safety participation than those from middle income groups (*F* = 2.67; *p* < 0.05). According to the results of the study, the mean scores for safety climate, safety performance, safety participation, and safety compliance showed no significant difference between male and female participants. Education, marital status and work experience had no role in the safety climate and safety performance.

Participants were also asked about the number of occupational accidents, occupational diseases, and exposure to verbal, physical and psychological violence in the last 2 years. Based on Anova tests, higher safety climate perceptions (*F* = 11.095; *p* < 0.001) and safety compliance scores (*F* = 5.002; *p* < 0.01) were found in employee groups that had no occupational accidents (52.8%) in the last 2 years. Similarly, safety climate perception (*F* = 14.751; *p* < 0.001) and safety performance scores (*F* = 2.979; *p* < 0.05) were higher in participants who had no occupational illnesses (34.1%) in the last 2 years. On the other hand, employees who had not experienced verbal, physical or psychological violence (29.7%) had higher safety climate perceptions (*F* = 21.459; *p* < 0.001) and safety performance scores (*F* = 3.310; *p* < 0.05).

### 4.3 The most common causes of occupational accidents and occupational diseases faced by employees

Employees were asked whether they had been involved in an accident at work in the last 2 years and, if so, what caused it. The results of the descriptive statistics are shown in Table 4.

**Table 4:** The most common causes of occupational accidents faced by employees.

Causes of occupational accidents*	*n*	%
I never had a workplace accident.	245	52.8
I was injured due to shaking in the ambulance.	85	18.3
I was injured because of an infected needlestick.	95	20.5
I had an injury due to carrying a patient.	78	16.8
I was injured due to a traffic accident.	74	16
I had a cutting/piercing injury.	71	15.3
I suffered an injury due to personal protective equipment.	11	2.3
Other	38	8.1

* Employees were told that they could select multiple choices.

The results of this study showed that the most common occupational accidents suffered by workers were needlestick injuries, shaking in ambulances, carrying patients, and injuries caused by cutting/cutting.

Participants were asked if they had suffered from a work-related disease in the last 2 years and, if so, the nature of the disease. The results of the descriptive statistics are shown in Table 5.

**Table 5:** The most common types of occupational diseases suffered by employees.

Types of occupational diseases*	*n*	%
I never had an occupational disease.	158	34.1
I had a herniated disc (lumbar disc herniation).	223	48.1
I suffered from insomnia	233	50.2
I suffered from mental illnesses. Mainly depression and anxiety.	168	36.2
I had a neck herniated disc.	93	20
I suffered from infectious diseases.	67	14.5
Other	7	1.5

* Employees were told that they could select multiple choices.

Table 5 shows that herniated discs, insomnia, depression, and anxiety are the most common occupational diseases among workers.

### 4.4 Path analysis results regarding the relationships between subdimensions of safety climate perception and safety performance of employees

**Figure 1:**
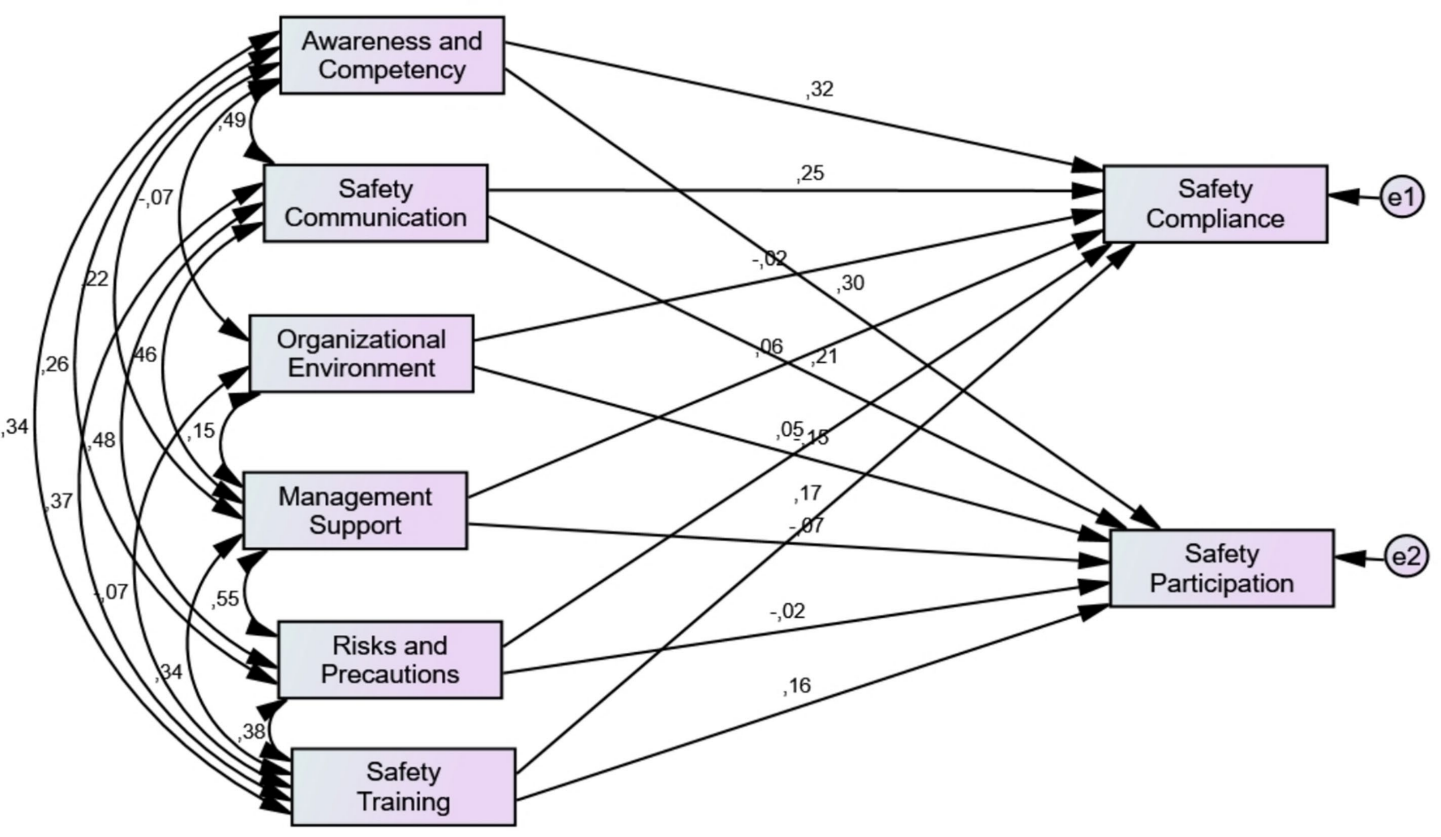
Path model between the subdimensions of the safety climate and the safety performance constructs. Model fit indices χ^2^/df = 2.066; GFI = 0.997; CFI = 0.997, AGFI = 0.960; RMSEA = 0.048; RMR = 0.016.

A path analysis was carried out to determine the relationships of the sub-dimensions of safety climate perception with the sub-dimensions of worker safety performance, and the results of the hypotheses are presented in Figure 1 and Table 6.

**Table 6:** Standardized path coefficients and results of hypothesis.

Path			B	SE	*β*	*t*	*p*	Hypothesis	Result
Safety compliance	⇐	Awareness and competency	0.402	0.052	0.323	7.688	**<0.001**	H_1a_	Supported
Safety compliance	⇐	Safety communication	0.230	0.044	0.246	5.223	**<0.001**	H_1b_	Supported
Safety compliance	⇐	Organizational environment	−0.021	0.035	−0.021	−0.584	0.559	H_1c_	Not Supported
Safety compliance	⇐	Management support	0.042	0.034	0.057	1.236	0.216	H_1d_	Not Supported
Safety compliance	⇐	Risks and precautions	0.043	0.041	0.048	1.050	0.294	H_1e_	Not Supported
Safety compliance	⇐	Safety training	0.148	0.035	0.173	4.223	**<0.001**	H_1f_	Supported
Safety participation	⇐	Awareness and competency	0.308	0.048	0.300	6.475	**<0.001**	H_2a_	Supported
Safety participation	⇐	Safety communication	0.159	0.040	0.207	3.987	**<0.001**	H_2b_	Supported
Safety participation	⇐	Organizational environment	−0.119	0.032	−0.150	−3.721	**<0.001**	H_2c_	Supported
Safety participation	⇐	Management support	−0.041	0.031	−0.068	−1.336	0.182	H_2d_	Not Supported
Safety participation	⇐	Risks and precautions	−0.015	0.037	−0.020	−0.394	0.694	H_2e_	Not Supported
Safety participation	⇐	Safety training	0.115	0.032	0.163	3.606	**<0.001**	H_2f_	Supported

S.E., standardized error.

Significance of *p*-level based on <0.05.

Figure 1 shows the paths between the subdimensions of the safety climate and the safety performance constructs. The results of the path analysis can be followed in Figure 1.

As shown in Figure 1, the indices CFI, χ^2^/df, GFI, RMSEA, AGFI, and RMR confirmed the unidimensional of the model constructs and were found to be satisfactory. Figure 1 shows that safety communication, safety competence and awareness and competence and safety participation are correlated with safety performance positively. On the other hand, organizational environment is negatively correlated with safety participation. All negative and positive pathways and significance of path coefficients between subdimensions of the safety climate and the safety performance constructs are explained in Table 6. Additionally, supported and unsupported results of the hypotheses of the research are presented in Table 6.

Based on the results of this study, some hypotheses were supported while others were not. According to Table 6, there is a significant positive relationship between awareness and competence (*β* = 0.300; *p* < 0.001), safety training (*β* = 0.163; *p* < 0.001) and safety communication (*β* = 0.207; *p* < 0.001), and employee safety compliance (*p* < 0.001). The results showed that the higher the level of communication and training in safety, the higher the safety compliance of workers in pre-hospital emergency. Furthermore, when pre-hospital emergency employees’ awareness and competence increased, they complied with safety rules more. Thus, H_1a_, H_1b_, H_1f_ are supported. On the other hand, organizational environment, management support, and risks and precautions are not significantly related to safety compliance (*p* > 0.05). Therefore, H_1c_, H_1d_ are not supported.

The study found a significant positive relationship between awareness and competence, safety communication, safety training, and employee participation in safety (*p* < 0.001). Therefore, H_2a_, H_2b,_ H_2c_ are supported. Another important finding of this study is the significant negative relationship of the organizational environment (*β* = −0.150, *p* < 0.05) with workers’ safety participation. Considering these findings, H_2f_ is supported, but H_2d_, H_2e_ are not supported.

### 4.5 Linear regression results of safety climate and safety performance of workers

To calculate the relationship between safety climate perception and employee safety performance, a linear regression was performed, and the results are shown in Table 7.

**Table 7:** Linear regression results of safety climate and safety performance of workers.

Dependent variable	Independent variable	B	SE	*β*	*t*	*p*
Safety performance	Constant	2.305	0.156		14.789	**<0.001**
	Safety climate	0.568	0.048	0.480	11.759	**<0.001**

Model *R*^2^ = 0.230; Adjusted *R*^2^ = 0.229; *F* = 138.264; *p* < 0.001.

Significance of *p*-level based on <0.05.

Table 7 shows that the perception of the safety climate in the workplace has a positive relationship with the safety performance of workers (*β* = 0.480; *p* < 0.001). The regression model was found to be statistically significant (*F* = 138.264; *p* < 0.001) and suggests that 22.9% of the variance in Safety Performance scores is explained by the model. Concerning this, it can be stated that pre-hospital emergency workers’ safety performance increased as their levels of safety climate perception increased. Thus, H_3_ is supported.

A multiple linear regression test was performed to determine the relationship between safety performance subdimensions and safety performance of pre-hospital emergency health workers and the results are shown in Table 8.

**Table 8:** Multiple linear regression results of safety climate and safety performance subdimensions of workers.

Dependent variable	Independent variable	B	SE	*β*	*t*	*p*
Safety performance	Constant	1.449	0.180		8.053	**<0.001**
	Awareness and Competency	0.355	0.043	0.347	8.250	**<0.001**
	Safety Communication	0.194	0.036	0.252	5.368	**<0.001**
	Organizational Environment	−0.070	0.029	−0.088	−2.396	**<0.05**
	Management Support	0.000	0.028	0.001	0.013	0.990
	Risks and Precautions	0.014	0.034	0.019	0.420	0.675
	Safety Training	0.131	0.029	0.187	4.547	**<0.001**

Model *R*^2^ = 0,416; Adjusted *R*^2^ = 0.409; *F* = 54,327; *p* < 0.001.

Significance of *p*-level based on <0.05.

Based on the findings shown in Table 8, Awareness and Competency (*β* = 0.347; *p* < 0.001) and Safety Communication (*β* = 0.187; *p* < 0.001) have a significant relationship with the Safety Performance of the employees. The results showed that the higher awareness and competency of employees, the safer they perform. Furthermore, when communication about safety increased in the workplace, pre-hospital emergency health workers behaved more safely. On the other hand, there is a significant negative relationship between Organizational Environment and Safety Performance of pre-hospital emergency healthcare workers (*β* = −0.088; *p* < 0.05). According to the results, when the negative conditions, such as time pressure or uncontrollable scenes decreased workplace safety, pre-hospital emergency workers tried to take control and behave more safely. This study found no significant relationship between Management support and Risks and Precautions and Safety Performance of the employees (*p* > 0.05).

When correlation coefficients between dependent and independent variables were calculated, it was seen that occupational safety performance score has a positive and significant relationship with awareness and competence (*r* = 0.550; *p* < 0.001), safety communication (*r* = 0.509; *p* < 0.001), management support (*r* = 0.250; *p* < 0.001), risks and precautions (*r* = 0.301; *p* < 0.001), safety training (*r* = 0.415; *p* < 0.001) and occupational safety climate (*r* = 0.480; *p* < 0.001). A negative and significant relationship was found between organizational environment and occupational safety performance (*r* = −0.157; *p* < 0.01). When the other dependent variables were considered awareness and competency was positively and significantly related with safety communication (*r* = 0,492; *p* < 0.001), management support (*r* = 0.212; *p* < 0.001) and risks and precautions (*r* = 0.262; *p* < 0.001) on the other hand it was negatively and significantly related to organizational environment (*r* = −0.104; *p* < 0.05). Safety communication was positively and significantly related to management support (*r* = 0.455; *p* < 0.001), risks and precautions (*r* = 0.578; *p* < 0.001) and safety training (*r* = 0.375; *p* < 0.001) but not significantly related to organizational environment (*r* = −0.071; *p* > 0.05). There was a negative relationship between organizational environment and safety training (*r* = −0.080; *p* > 0.05), but it was not significant. There was a positive significant relationship between risks and precautions was and management support (*r* = 0.339; *p* < 0.001).

## 5 Discussion

### 5.1 Safety performance, safety climate and socio-demographics of employees

This study revealed that the perception of workplace safety climate and safety performance of pre-hospital emergency health services employees vary based on sociodemographic variables. Older employees were found to have a higher perception of safety climate in the workplace. This can be attributed to more work and professional experience. Younger individuals, on the other hand, may exaggerate even minor dangers due to their inexperience, which may cause them to feel less safe. More experienced older individuals may previously faced hazardous situations several times, so they know the necessary precautions and may be prepared for potential risks. Similarly, Gümüş et al. reported lower safety climate perception among young groups in their study conducted in the mining sector (39). Gümüş et al. attributed the low perception of safety climate among young employees to their high expectations in the workplace and being aware of their rights and responsibilities of management. On the other hand, Uğur et al. (40) found no significant correlation between age and safety climate perception in their study, which combined data from various sectors. Additionally, Aydın et al. (41) reported similar results in the study performed with nurses. They reported that workers behave safely at various ages. In the construction sector, He et al. (42) implied that the younger and older groups’ safety climate perception was not significantly different. The findings of the current study conflict with these studies.

The current study found no difference between the groups in terms of safety climate perception by gender variable. This may be attributed to the uniqueness of pre-hospital emergency health services that never tolerate safety negligence. This finding shows that safety climate and safety performance are perceived similarly by all female and male employees, regardless of gender. The present study supports the findings of Uğur et al. (40), who did not report a significant difference between male and female groups in terms of safety climate perception. Also, He et al. found similar results in their study conducted in the construction sector. On the other hand, it contradicts that of Aydın et al. (41), who found higher scores of safety climate perception in female groups. The current study also contradicts Öğüt and Akın (43), reporting higher safety climate perception scores among male groups. In their study, female employees working in units containing radiation fields in hospitals perceive a lower safety climate than male employees. This may be because women are more sensitive to health-related issues and believe that health problems will increase in radiation areas.

In the current study, the perception of safety climate and safety performance among employees working in pre-hospital emergency health services did not differ by marital status. Similarly, Gümüş et al. (39) found no significant difference between married and single employees in the study conducted in the mining sector. The mining industry poses occupational health risks as much as the health sector, and every employee, regardless of whether they are single or married, has a similar perception of safety at work. The similar results found in the current study may be due to this reason. Kılıç and Acar (44) reported that work-related anxiety and stress stemming from work workplace were not significantly different among emergency healthcare workers by marital status. Also, findings of Uğur et al. (40) are in alignment with the current study.

Considering income status, participants in the high-income group showed higher safety participation. This can be attributed to their better working conditions, more access to security applications, or a higher tendency to attach more importance to these issues. These findings align with those of Gümüş et al. (39), who implied that employees from higher income groups may be more educated and working in higher-level career jobs. So, they may play key roles in the perception of safety climate of the workplace. On the other hand, Aydın et al. (41) did not find a significant difference in safety climate perception between income groups. The current study conflicts with the study of Aydın et al., who performed their study in a more homogeneous group consisting of nurses.

Given the employees’ education levels, those with lower education had higher mean scores in the risks and precautions sub-dimension of the safety climate. The groups with lower education levels primarily consist of drivers. Considering that these groups perform the most dangerous tasks and are exposed to many risks in traffic, the results are plausible. Those individuals feel the risks in the workplace more and believe that more attention should be paid to precautions. These findings are consistent with those of Öğüt and Akın (43), who performed their study with employees working in units containing radiation fields of hospitals. This might be because employees from lower-educated groups are technicians who are working in areas receiving intense radiation.

This study found that the perception of safety climate did not differ by work experience. These findings contrast with those of Aydın et al. (41), who found lower safety climate perception in nurse groups who had more work experience. On the other hand, Çakmak and Tatlı (45) reported higher safety climate scores in healthcare workers who had higher work experience. However, Gümüş et al. (39) and Öğüt and Akın (43) yielded similar results to this study. They reported that employees from different work experiences showed similar safety climate perception scores. Whether they all work in the health sector, whether they are experienced or inexperienced, the structure of the job and the place of work have different effects on the perception of the safety climate of the employees.

### 5.2 Occupational accidents, diseases, and exposure to violence among employees

The results of this study determined that almost half of those working in pre-hospital emergency health services had at least one occupational accident within the last 2 years. In addition, half of them had at least two occupational diseases or work-related injuries during the same period. Those individuals are at high risk in terms of occupational health and safety. Some studies conducted with healthcare workers in Türkiye revealed that healthcare workers are always at risk of violence (46, 47). Similarly, the results of this study showed that those working in pre-hospital emergency health services are at high risk in terms of occupational health and safety. The prevalence of occupational accidents and diseases highlights the inadequacy of the current measures, underlining that working conditions should be improved. These findings are consistent with the studies of Kızıl (48), Sarıkahya et al. (49), and Gülen et al. (50). Studies conducted by Probst and Estrada (51), McGhan et al. (30), and Huang et al. (16) support the findings of the present study, reporting that employees with a high perception of safety climate are exposed to fewer accidents.

Half of the employees reported having been subjected to both verbal and physical violence in the last 2 years. Being the first responders to emergencies, pre-hospital emergency healthcare workers are frequently subjected to violence from patients and their relatives. In cases of serious trauma, both patients and their relatives often attribute any delays or mishaps to these workers. This tendency reveals the need to take more effective measures to address violence in the workplace. These findings align with those of previous studies conducted in Türkiye (49, 50, 52, 53).

The participants were asked to identify the most common causes of injuries, with infected needlestick injuries, injuries from sharp and piercing tools, injuries due to shaking inside the ambulance, and accidents during transport of patients being the most frequently reported. Since they must prioritize patient safety in the ambulance, employees often place their safety at risk. Furthermore, the high speed of ambulances exposes employees to various risks during sudden maneuvers, and the urgency of responding to emergencies may lead to the neglect of necessary precautions. In addition, they sometimes must lower patients from the upper floors of buildings with only two employees, which can exceed safe carrying capacities. Their physical characteristics also make it difficult to carry such weights. These findings are consistent with those of Önal (53), Sönmez et al. (54), and Kılıç et al. (44).

When participants were asked about the most common occupational diseases they experienced, half reported having sleep disorders. Extended shifts affect the daily lives and health of employees. Those who experience sleep disorders reported difficulty solving this problem even on their days off. At least half of the employees reported neck and back pain due to lifting heavy loads. They stated that emergency response equipment is heavy, and they sometimes carry it to upper floors without an elevator. When patient transportation is added to this, employees are exposed to very serious ergonomic risks. Mental illnesses, particularly depression and anxiety, were reported as the most common complaints among employees. Harsh working conditions, the obligation to respond to patients in emergencies, time pressure, and the intensity of work in natural and unnatural disasters have very serious negative effects on the mental health of employees. These findings revealed that those working in pre-hospital emergency health services are exposed to serious occupational risks. Sönmez et al. (54) and Kılıç et al. (44) reported similar results to the current study.

### 5.3 The association of safety climate sub-dimensions with employees’ safety performance

Safety climate perception has multiple dimensions. These include the management’s approach to safety, safety training, the communication of employees with each other and with management regarding safety issues, and the risk levels and precautions taken by employees. The most critical element included in the safety climate is the risks and safety factors that arise from the nature of the job, referred to as the organizational environment. As mentioned in the topics discussed below, the perception of safety climate greatly impacts employees’ safe behaviors, compliance with safety measures, and sharing and participating with their colleagues and managers regarding safety. The higher the safety climate in a workplace, the more likely employees are to exhibit safe behaviors.

According to the study results, the safety climate perception among employees working in pre-hospital emergency health services has a positive relationship with safety performance. Factors such as management’s attitudes in the workplace, the safety training received by the employees, and the safety communication in the workplace positively affect the safe behaviors of employees. In line with this study, previous studies examining the relationship between the safety climate in the workplace and safety performance also revealed that the safety climate directly affects safe behaviors in the workplace positively (24, 27, 38). Similar results were observed in studies conducted in Türkiye. Çakmak and Tatlı (45) emphasize that as the perception of safety climate improves among healthcare professionals, safety performance also increases. Kara and Oğuzöncül (55) revealed a positive relationship between the perception of safety climate and safety performance in their study with doctors, midwives, and nurses. Research by Pousette et al. (56), Zhou et al. (24), Griffin and Neal (21), Zohar and Luria (57), and Brondino et al. (58) also reported the positive effect of safety climate on safety participation and safety compliance. These studies also align with the current research.

The findings revealed that the most important sub-dimensions of safety climate positively related to safety performance are awareness and competence, safety communication, and safety training. These results are supported by studies conducted with healthcare workers by Aydın and Seren (41) and Çakmak and Tatlı (45), both of which emphasized the effect of safety training on safe behaviors. Other studies underscoring the impact of safety communication on safety performance reached similar conclusions to the current study (29, 39, 45, 59). Gümüş et al. (39) stated that when workers can freely express their problems or opinions in the workplace, they obey the rules and put extra effort to contribute to occupational health and safety in the organization. Zohar and Luria (57) also reported that efficient communication in the workplace contributes to the safety behaviors of the employees.

The least related factor to safety compliance and safety participation of the workers is managerial support. This result showed that the safety performance of employees in pre-hospital emergency services is not driven by their expectations from management. On the other hand, several studies from the literature show conflicting results. Gümüş et al. (39) found that management’s safety commitment was the most significant element contributing to the safety performance of employees in marble factories. Moreover, several studies showed that management’s support for the employees is the most important factor in safety behaviors of the employees (18, 22, 27, 57). In these studies, it was emphasized that when managements focus on occupational health in the workplace, employees feel themselves valuable. Therefore, the employees behave safely, obey the rules voluntarily, and help other coworkers. In this study pre-hospital emergency healthcare workers, regardless of the management’s attitudes, behave safely and obey the rules. Undoubtedly, the support of managers in the workplace, their interest in safety issues, and their prioritization of taking precautions before accidents occur affect safety behaviors in all workplaces, but pre-hospital emergency health workers are highly aware of their responsibilities and duties.

This study found no significant relationship between Risks and Precautions subdimension of safety climate and safety compliance and safety participation of the employees. On the other hand, Arezes (60), Kaouabenon (61) and Kauabenon et al. (62) reported a positive relationship between perceived risk and safety behavior of workers. The studies summarized that risk exposure is a triggering element for the employees’ concern about safety issues and the possibility of employees facing an accident at any time in the workplace, or the precautions taken against these factors affect their own work safety behaviors. Considering that these studies were conducted in different sectors and the priorities and levels of responsibility of pre-hospital emergency healthcare workers are considered, it is easier to understand these different results.

Another finding of this study is the negative and significant relationship between the organizational environment and the safety participation of the employees. Pre-hospital emergency healthcare workers believe that the nature of their work poses an obstacle to their safe behaviors. Responses to the questions within the organizational environment sub-dimension of the safety climate revealed that they sometimes cannot take safety precautions due to the urgency and high volume of tasks. Considering the nature of emergency health services, time pressure, and urgency of the task, this conclusion seems plausible. Time pressure and stress can lead employees to exhibit unsafe behaviors. While trying to cope with these negativities that come from the nature of the job, they take control by ignoring the difficulties they have and try to exhibit safe behaviors as best they can. Some studies conducted in occupations that expose one to work pressure found that job pressure or struggling against time briefly negativities in the workplace affected safety behaviors positively (9, 63, 64). On the other hand, Öz and Lajunen (65) and Wu et al. (66) reported that work pressure or emergency response did not affect employees’ safety behaviors and these findings are not in alignment with this study. These results may be due to the fact that the sectors in which the research was conducted were not health. Considering the nature of pre-hospital emergency health services, time pressure, and urgency of the task, the findings of this study seem plausible. Regardless of the circumstances, those working in pre-hospital emergency health services are aware of the seriousness of the job and the importance of health, and they try to act accordingly. To this end, regardless of the circumstances, they strive to perform their jobs to the best of their ability and take safety precautions in emergencies.

## 6 Conclusion and future directions

This study, which investigated the association of safety climate perception in the workplace with the safety performance of employees working in pre-hospital emergency health services, yielded important findings. According to the results, the majority of the participants had been subjected to violence, had an occupational accident, and had an occupational disease. These findings supported the data reports of World Health Organization, which defined the health sector among the most dangerous professions (7).

High-level risks and dangers exist across all healthcare fields, with pre-hospital health services being at the highest risk. In pre-hospital emergency services, every location is a potential work area. In most cases, precautions are not taken when the team arrives, and, in some cases, it is impossible to take precautions. Therefore, a large part of the responsibility falls on the employees. The workplaces of pre-hospital emergency healthcare workers can range from a traffic accident or fire to a collapsed building during an earthquake, environments impacted by pandemics, sites of explosions, or flooded places. That is why employees pay the highest attention to the safety of themselves and their patients. These findings suggest that more precautions should be taken against workplace risks, and more training should be provided for employees. The participants recognize the significant benefits of safety training in addressing dangers in the work environment. As a result, the duration and variety of personnel training should be enhanced. The use of protective equipment and adherence to proper handling rules are also among the most crucial factors to be addressed during training. The participants also reported frequently experiencing disorders such as insomnia and anxiety. Psychological support for pre-hospital emergency health service workers, who are exposed to various traumatic events and people due to the nature of their work, can also help improve their mental health.

Improving the physical environment in pre-hospital health services is crucial for occupational safety. The physical environment of the ambulance stations and ambulances should be monitored and further improved. Ambulance drivers, who perform one of the most difficult jobs, must be careful and fast at the same time during transportation. They are responsible for ensuring the well-being of both the patient and the staff. As a result, they are the group most exposed to the risks arising from the organizational environment. Their occupational risks should be minimized through proper work scheduling and organization. In addition, there should be efforts to maintain ambulances and to make people more empathetic toward ambulance drivers.

Working hours are also among the most important obstacles to safety. Employees working in 24 h shifts suffer from sleep deprivation and other reasons, making them more vulnerable to dangers. The workload of this group, who do not have sleeping and eating patterns, should be reduced. The participants stated that adverse conditions stemming from the organizational environment reduce safety performance. Nevertheless, they are aware that they need to be much more careful due to factors such as time pressure, ensuring the safety of the patient and themselves, and the difficulties of taking precautions against workplace hazards. To eliminate occupational health and safety issues in pre-hospital emergency health services, a shift system that reduces workload should be established. Ensuring healthier and safer stations, increasing the number of personnel, and implementing more efficient and effective work plans can improve the working conditions of pre-hospital emergency healthcare workers.

The study results indicated that increasing the safety climate in the workplace plays an important role in the safe behavior of employees. Therefore, both managers and employees must contribute to increasing the safety climate in the workplace, whether in stations, ambulances, or at the scene. Additionally, both the Ministry of Health and other official institutions should work on enacting new laws and regulations to ensure occupational health and safety practices in pre-hospital emergency health services.

### 6.1 Limitations of the study

This study had several limitations. For instance, all the study variables were collected with self-reported measures. By only incorporating self-report survey data, common method bias may have affected these results. Additionally, this study is cross-sectional and focuses on a particular moment in time. Hence, no causal inferences can be made for the population; such a statement of causal inferences requires the collection of longitudinal data. So, future studies are recommended to use a longitudinal research design to detect variations over time. Another limitation of this study is its low generalizability. There are seven regions in Türkiye. Working conditions and characteristics of each region may vary. The region where this study was conducted may have unique characteristics, which may affect the results of the study. Therefore, the results obtained in this study cannot be generalized to the entire country. The results will become more generalizable with the implementation of similar studies in different regions of the country. Although the study achieved the targeted sample size, it would have been more efficient to include more employees. Furthermore, pre-hospital emergency health services workers do not have the opportunity to work in a fixed location and timeframe. Their workspace varies across all incident locations, and their work schedules vary depending on their shift schedule. This study did not employ a specific sampling method, aiming to reach the entire study population. Therefore, all stations included in the study population were visited. However, due to the nature of the work, it was not possible to reach all employees.

## Data Availability

The raw data supporting the conclusions of this article will be made available by the authors, without undue reservation.
